# Supplementary material for: Inhibition of IRAK1 Is an Effective Therapy for Autoimmune Hypophysitis in Mice
Source: Int J Mol Sci. 2022 Nov 29;23(23):14958. doi: 10.3390/ijms232314958 (PMC9738236; doi:10.3390/ijms232314958)
Supplement: Supplementary file 1 [file ijms-23-14958-s001.zip › ijms-1992055-supplementary.pdf]

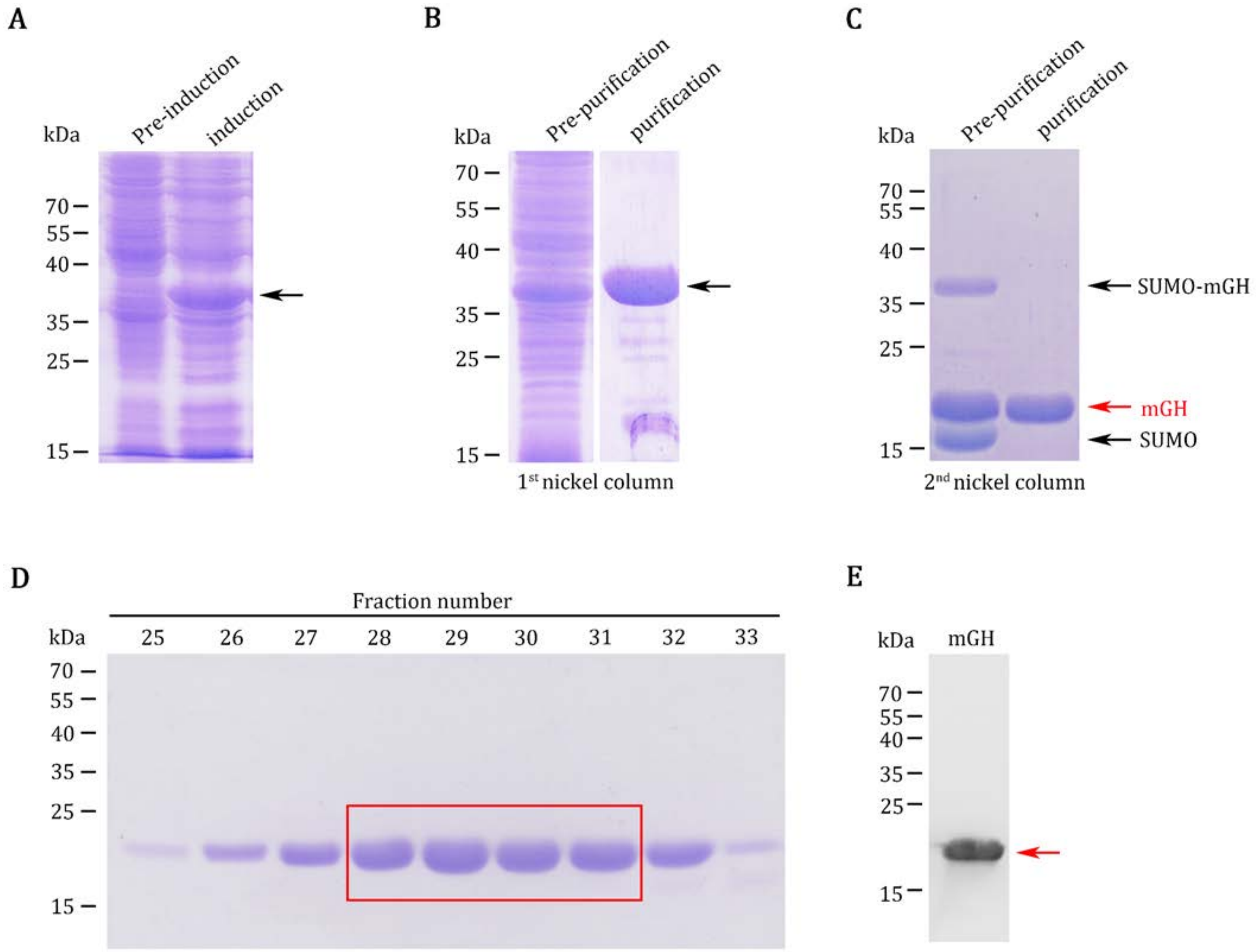

Supplementary Figure S1. Production and purification of recombinant growth hormone protein (mGH). SHuffle T7 express competent cells were transformed by an expression vector encoding the His-tag-SUMO peptide-mGH. **(A)** Expression of His-tag-SUMO peptide-mGH fusion protein (37 kDa) by the competent cells was induced by IPTG (arrow). **(B)** His-tag-SUMO peptide-mGH fusion protein was purified from the lysates of competent cells by a nickel column. **(C)** His tag-SUMO peptide-mGH fusion protein was digested with SUMO protease to remove His-tag-SUMO peptide from mGH by a nickel column. **(D)** mGH was further purified to near homogeneity by a S-100 sephacryl size-exclusion column. Fractions enclosed by the red rectangle were used for immunization. **(E)** Purified mGH was analyzed by western blots using polled sera of EAH mice previously induced by mGH.

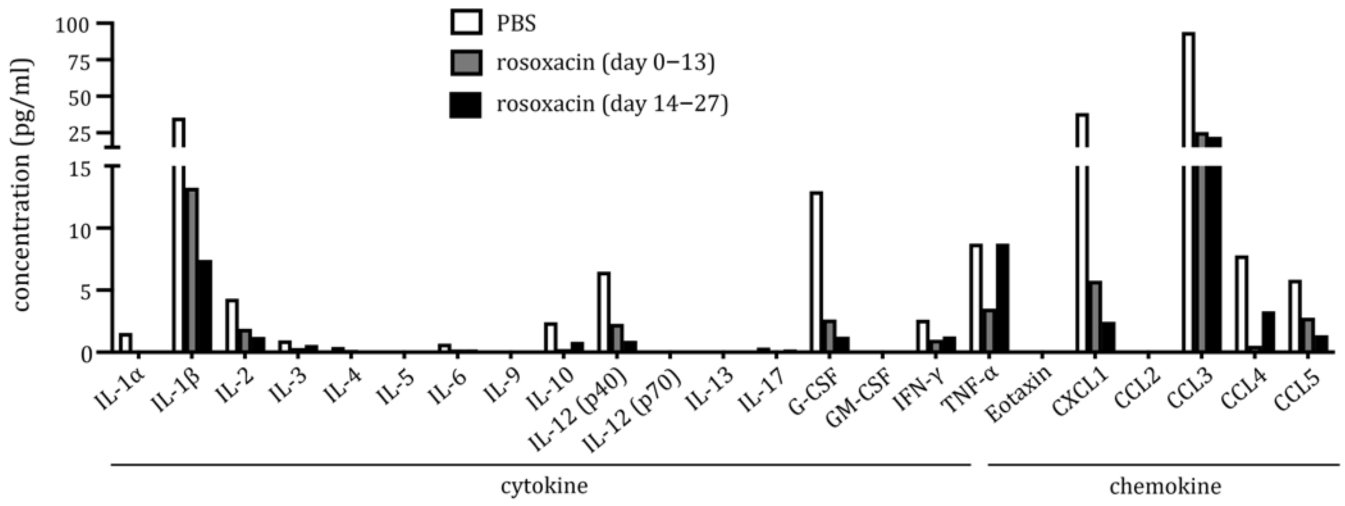

Supplementary Figure S2. Effects of rosoxacin on secretion of 23 different cytokines and chemokines by lymphocytes in vitro. Lymphocytes isolated from deep cervical lymph nodes of EAH mice received different treatments were culture for 96 h. The levels of cytokines and chemokines secreted by lymph node cells were determine by a multiplex cytokine assay. Cytokines and chemokines tested in the assay included IL-1α, IL-1β, IL-2, IL-3, IL-4, IL-5, IL-6, IL-9, IL-10, IL-12 (p40), IL-12 (p70), IL-13, IL-17, G-CSF, GM-CSF, IFN-γ, TNF-α, Eotaxin, CXCL1, CCL-2, CCL-3, CCL4, and CCL5.
