# Supplementary figures and images for: Inhibition of IRAK1 Is an Effective Therapy for Autoimmune Hypophysitis in Mice
Source: Int J Mol Sci. 2022 Nov 29;23(23):14958. doi: 10.3390/ijms232314958 (PMC9738236; doi:10.3390/ijms232314958)

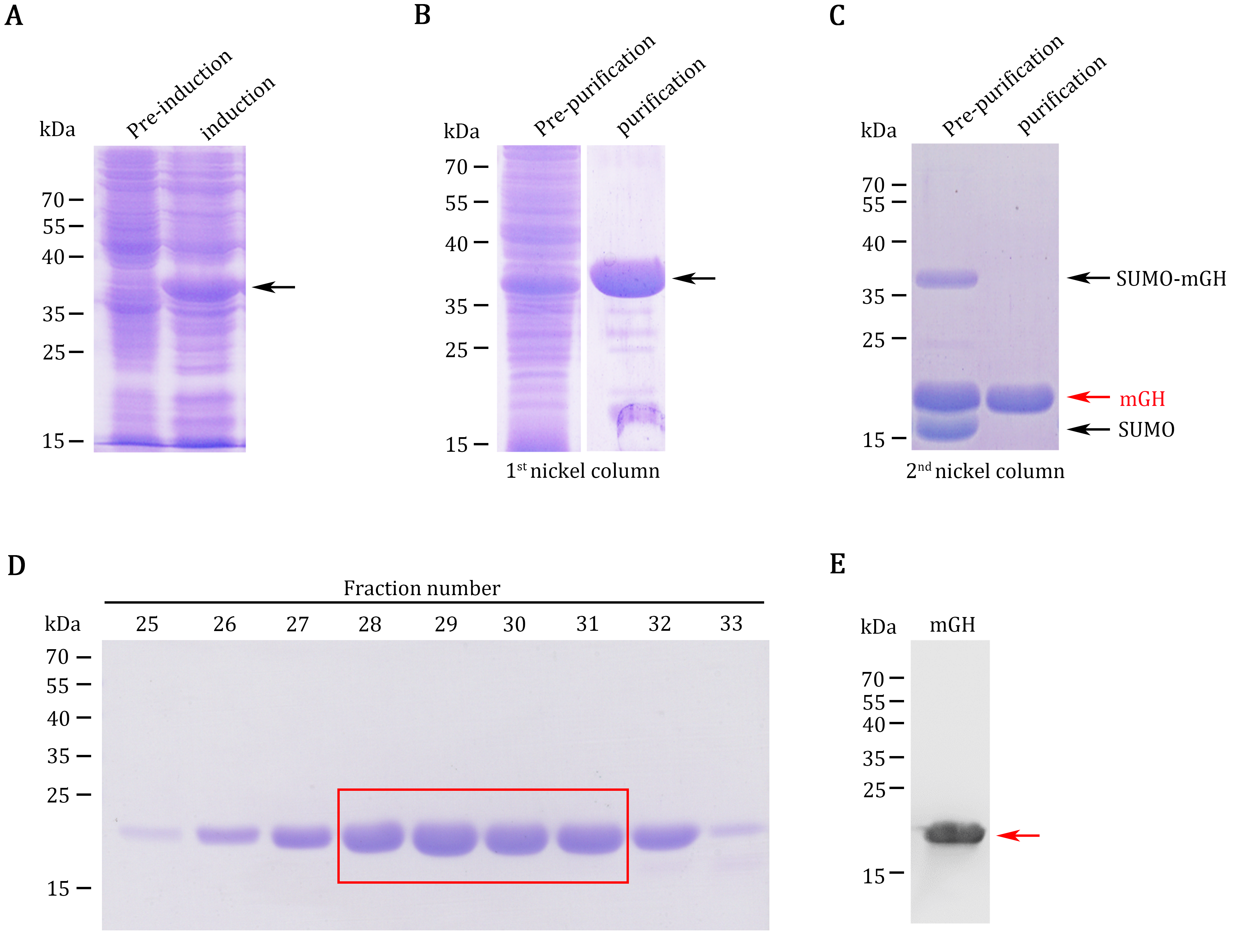

Supplement: Supplementary file 1 [file ijms-23-14958-s001.zip › Supplementary Figure S1.tif]

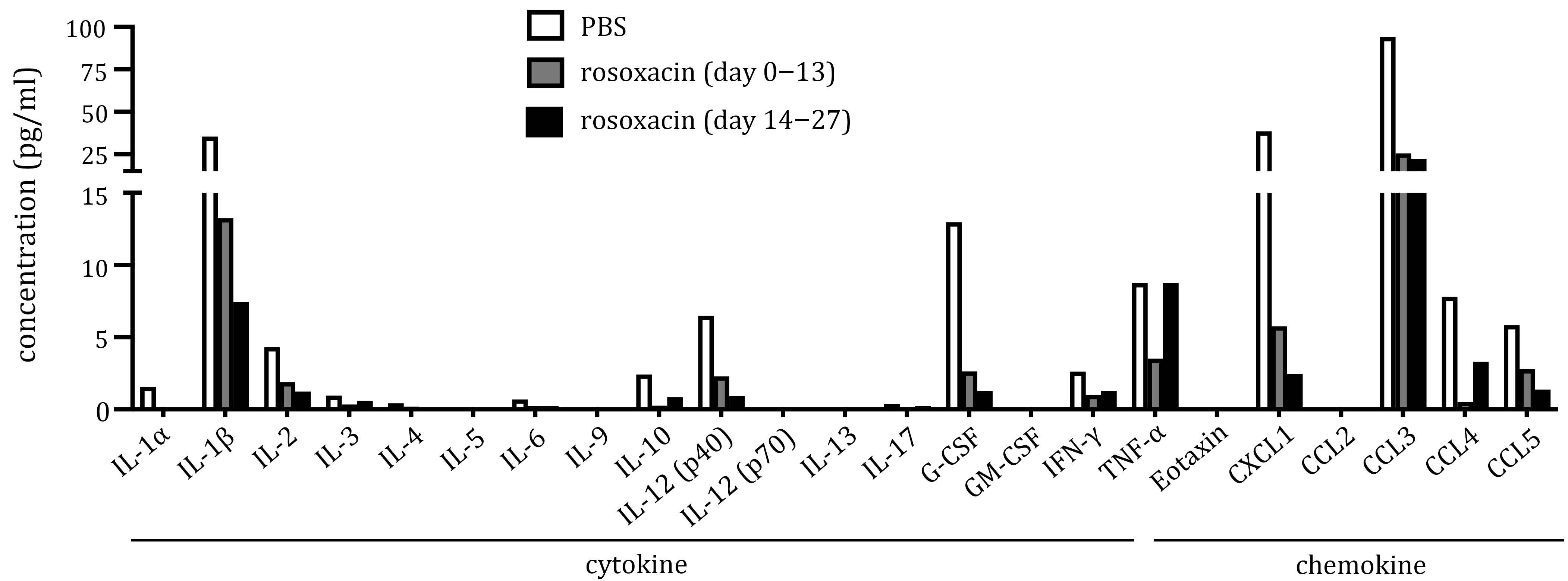

Supplement: Supplementary file 1 [file ijms-23-14958-s001.zip › Supplemetary Figure S2.tif]
